# Case Report of snare-assisted coaxiality optimized technique during valve deployment in patient with pure aortic regurgitation

**DOI:** 10.3389/fcvm.2024.1383264

**Published:** 2024-05-09

**Authors:** Jiwei Yu, Run Du, Fenghua Ding, Ruiyan Zhang, Zhengbin Zhu

**Affiliations:** ^1^ Department of Cardiology, Rui Jin Hospital, Shanghai Jiao Tong University School of Medicine, Shanghai, China; ^2^ Cardiovascular Research Institution, Shanghai Jiao Tong University School of Medicine, Shanghai, China

**Keywords:** aortic valve stenosis, snare catheter, snare-assisted coaxiality optimized technique, TAVR, transcatheter valve replacement

## Abstract

In high-risk patients with pure native aortic regurgitation (PNAR), transcatheter aortic valve replacement (TAVR) remains an off-label intervention. Due to anatomical variations in the aortic root and technical challenges unique to PNAR, the transfemoral approach (TF-TAVR) requires continued accumulation of experience and technological refinement. In this context, we successfully and safely performed a snare-assisted TF-TAVR procedure for a patient with PNAR, characterized by significant aortic angulation. We introduced an innovative technique termed “snare-assisted coaxiality optimized technique” (SACOT) during valve deployment. SACOT played a crucial role in optimizing valve positioning, enhancing coaxiality, and achieving the ideal implantation depth for PNAR. Post-procedure assessments demonstrated stability and the absence of paravalvular regurgitation (PVR).

## Introduction

**Figure 1:**
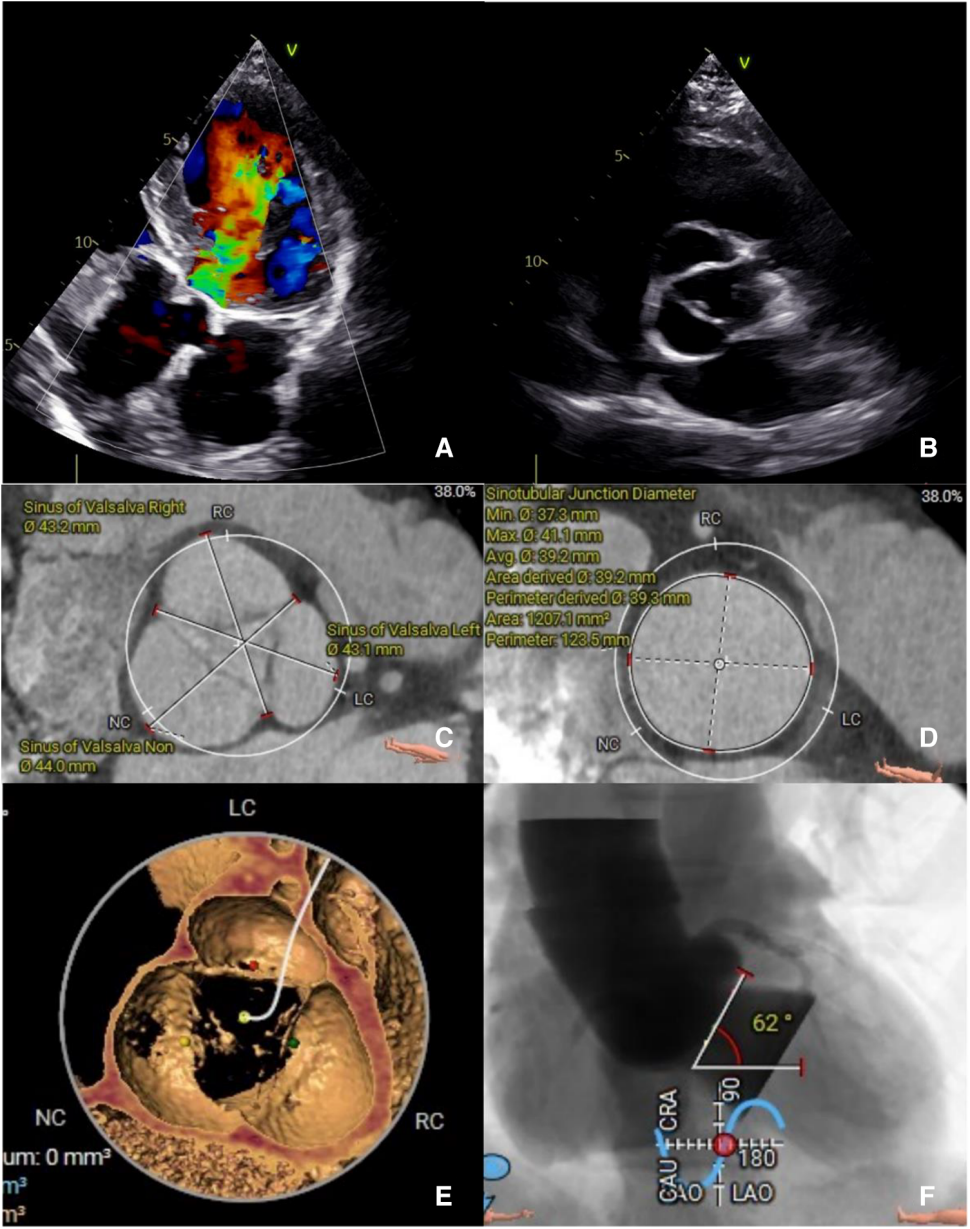
(**A**) The preoperative transthoracic echocardiogram in diastole showing severe AR and (**B**) absence of annulus and leaflets calcification. A preoperative computed tomography evaluation (**C,D**) showing a large sinus of Valsalva and sinus tubular junction. (**E**) No annulus of leaflets calcification. (**F**) Aortic angulation.

We describe a case of an 84-year-old male presenting with intermittent chest tightness and shortness of breath, with a significant exacerbation of symptoms over the past 2 weeks. He has a history of diabetes mellitus and chronic lung disease and underwent surgery for a pulmonary abscess previously. Additionally, he has a long-standing history of heavy smoking. Upon examination, his blood pressure measured 174/47 mmHg, heart rate was 74 bpm, and oxygen saturation was 92% on room air. Physical examination revealed normal respiratory sounds, diastolic murmur, and mild pedal edema. Screening of the preoperative laboratory values revealed elevated levels of N-terminal pro-brain natriuretic peptide (1,065 pg/ml; normal range, 5–738 pg/ml) and elevated creatinine (1.41 mg/dl; normal range, 0.7–1.29 mg/dl) level. Transthoracic echocardiography (TTE) was performed, revealing severe aortic regurgitation with a volume of 64 ml/beat, accompanied by mild mitral and tricuspid regurgitation. The left ventricular ejection fraction was 62%, the left ventricular end-diastolic diameter (LVEDd) was 62 mm, and the end-systolic diameter was 41 mm. Electrocardiography revealed sinus rhythm and increased QRS voltage (Figure 1).

After a thorough discussion with the heart team, we opted to proceed with a transcatheter aortic valve replacement (TAVR) using a 30 mm VitaFlow Liberty valve (MicroPort, Shanghai, China). This decision was influenced by the patient's Society of Thoracic Surgeons score, evaluated at 7.78%. Additionally, the patient declined surgical aortic valve replacement or transapical TAVR. Currently, there are no TF-TAVR devices specifically designed for PNAR available on the market in China. The VitaFlow transcatheter heart valve (THV), characterized by a straight cylindrical stent, enhances stability by increasing friction with surrounding tissues, while its external skirt serves to prevent PVR. Furthermore, its safety and efficacy have been confirmed (1).

A computed tomography (CT) angiography revealed a dilated sinus of Valsalva, with an average diameter of 43.4 mm, and a sinotubular junction (STJ), with an average diameter of 39.2 mm. No calcification was observed in the annulus or leaflets, with the aortic annulus area measuring 489.7 mm² and the perimeter measuring 79.6 mm. The left ventricular outflow tract (LVOT) perimeter was 85 mm, and the maximal ascending aorta diameter was 44.9 mm. Assessment of the coronary arteries and the arterial iliofemoral system anatomy indicated suitability for the TAVR procedure. Notably, the aortic angulation was 62°, which increases the surgical complexity for a patient with PNAR (Table 1, Figure 1).

**Table 1:** Pre-procedure computed tomography measurements of the aortic root.

Annulus (Systole)		Annulus (Diastole)		LVOT		Sinus of Valsalva			STJ
Perimeter (mm)	Area (mm^2^)	Perimeter (mm)	Area (mm^2^)	Perimeter (mm)	Area (mm^2^)	Diameter (mm)			Perimeter (mm)
						NCC	RCC	LCC	
79.6	489.7	79.3	473.9	85.0	528.5	43.1	43.2	44.0	123.5

LVOT, left ventricular outflow tract; NCC, non-coronary cusp; RCC, right coronary cusp; LCC, left coronary cusp; STJ, sinotubular junction.

**Figure 2:**
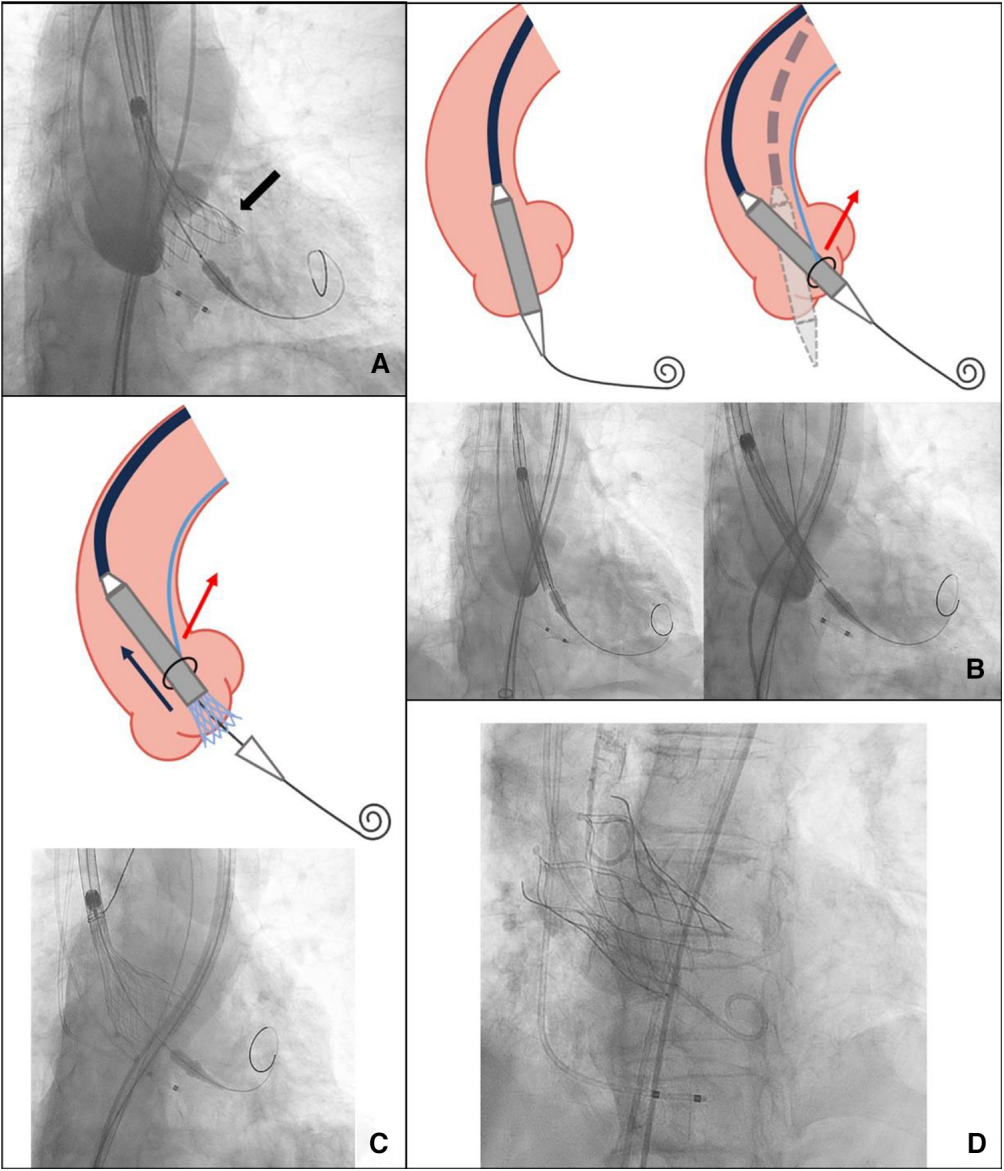
(**A**) An excessively deep implantation of the valve in the left coronary sinus (black arrow). (**B**) The coaxiality with and without SACOT. (**C**) SACOT continuously optimizing THV coaxiality during deployment. (**D**) Successful implantation. SACOT, snare-assisted coaxiality optimized technique during valve deployment.

The procedure was conducted under general anesthesia with guidance from a transthoracic echocardiogram. A pigtail catheter was placed in the sinus of Valsalva to serve as an annular landmark. The THV was carefully advanced until reaching the aortic annulus and was deployed under rapid ventricular pacing at 180 beats/min. Despite our attempts to position the valve as high as possible, the lack of coaxial alignment led to an excessively deep implantation of the valve in the left coronary sinus (Figure 2A).

**Figure 3:**
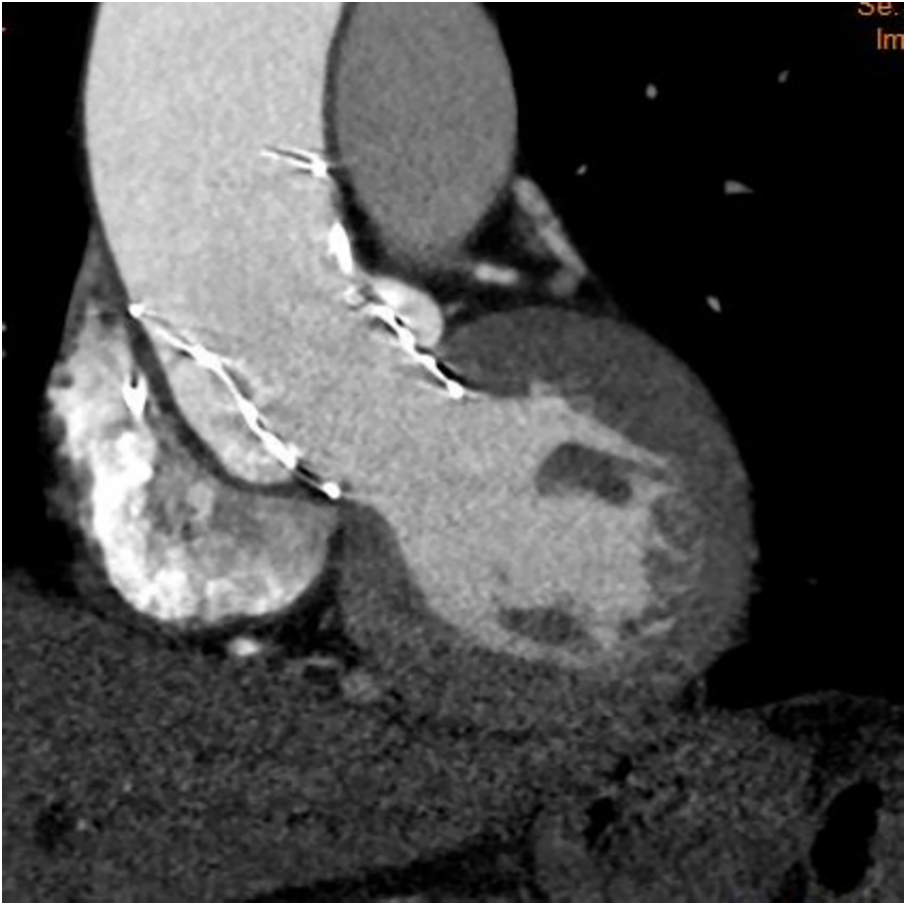
The follow-up computed tomography showing a will-positioned THV.

Therefore, we decided to pre-place a snare. The snare system, with a loop diameter of 20 mm and a 90° angle, was employed. A 4-F sheath was advanced through the ipsilateral femoral artery, with the prosthesis captured at the level of the descending aorta. The snare captured the distal fifth of the delivery capsule. During the second deployment, we used an unconventional approach by continuously pulling the prosthesis during deployment to enhance coaxiality until it reached the stable position. This led to a more optimal implantation depth below the left coronary sinus (Figures 2B–D). After the TTE assessment, no apparent perivalvular leak was observed, and there was no impact on the anterior leaflet motion of the mitral valve. We withdrew the snare catheter and released the prosthesis completely under rapid pacing at 120 beats/min to ensure valve stability. Upon a follow-up TTE before discharge, the LVEDd had decreased to 56 mm, and no residual AR was observed. The CT scan confirmed proper positioning and normal function of the THV (Figure 3).

Compared to AS, patients with PNAR often present with annular and LVOT dilation. The absence of calcified regions for anchoring, along with pathological dilation of the aortic valve annulus and ascending aorta, creates challenges in THV positioning and anchoring (2). Consequently, PNAR patients exhibit lower success rates, higher rates of valve-in-valve implantation, paravalvular leaks, migration, and the need for pacemaker implantation (3, 4).

Previously, case reports and case series have described snare-assisted TAVR as a solution for patients with severely tortuous or calcified aortas to avoid vascular complications (5). It has also served as a bail-out technique to facilitate THV passage through the aortic valve in cases with horizontal aortas or severely stenotic aortic valves. Typically, the operator withdraws the snare catheter once the prosthesis is in the correct position. In this patient, the proximal ascending aorta width measured 44.9 mm, with an aortic angulation of 62°. Conventional self-expanding valve deployment in such cases may result in poor coaxiality and excessive implantation depth, potentially resulting in paravalvular leaks or valve migration. We attempted a novel technique, which we named the “snare-assisted coaxiality optimized technique” (SACOT) during valve deployment. After the prosthesis was sent to the correction position, the snare catheter was not withdrawn. In cases where continuous adjustment of coaxiality is necessary during valve deployment, the snare can capture the prosthesis in the distal fifth and maintain the necessary tension to optimize the coaxiality during the deployment process until the valve is released into a stable position.

Currently, there are dedicated devices for PNAR, most of which are still in the clinical trial stage. The Trilogy Valve, which has received a CE mark, has demonstrated excellent performance in patients with PNAR due to its catheter deflection and locator technology, partially addressing the issue of coaxiality. However, dedicated devices for AR are not available in many regions. Therefore, under such circumstances, utilizing SACOT presents an alternative solution.

## Data Availability

The raw data supporting the conclusions of this article will be made available by the authors, without undue reservation.

## Supplementary material

Supplementary Video 1 Continuous optimization of coaxiality during the release process with SACOT.

Supplementary Video 2 Valve positioning and coaxiality adjustment assisted by SACOT.

Supplementary Video 3 Final result.
